# Driving impairment in patients with movement disorders: examining the Baylor driving questionnaire by objective driving assessment

**DOI:** 10.1016/j.prdoa.2026.100429

**Published:** 2026-02-10

**Authors:** Abhishek Lenka, Ruosha Li, Karim Makhoul, Alan Gonzalez, Rory D. Mahabir, Joseph Jankovic

**Affiliations:** a Parkinson’s Disease Center and Movement Disorders Clinic, Department of Neurology, Baylor College of Medicine, Houston, TX, USA; b Department of Biostatistics and Data Science, School of Public Health, The University of Texas Health Science Center at Houston, TX, USA

**Keywords:** Driving, Movement disorders, Parkinson’s disease, Tourette syndrome, Dystonia

## Abstract

**Background:**

The development of a screening tool to identify driving impairment in patients with movement disorders is an unmet need.

**Objective:**

To validate Baylor Driving Questionnaire for Movement Disorders (BDQMD) by objective driving assessment (ODA).

**Methods:**

In this cross-sectional study, 142 patients with various movement disorders completed the 10-item BDQMD and of those, 25 completed ODA using the driver performance analysis system (DPAS).

**Results:**

The mean total BDQMD score for the whole cohort was 14.7 ± 5.6 (range 10–47). Patients who underwent ODA had a mean BDQMD score of 12.8 ± 2.9. Four had minimum driving skill, 20 had average, and 1 was highly skilled. Total BDQMD negatively correlated with overall DPAS score (r = -0.45, p = 0.025) and the score reflecting the traffic risk analysis (r = -0.41, p = 0.041).

**Conclusion:**

BDQMD total score in this pilot study correlated well with the performance in ODA. Additional validation studies are warranted to explore its potential as a screening tool for driving impairment.

## Highlights

• Driving is an essential skill for independence and daily convenience.
• Several movement disorders can significantly impair driving ability.
• Currently, there is no standardized questionnaire to screen patients for driving impairment.
• This study evaluates the Baylor Driving Questionnaire in patients with movement disorders.

## 1 Introduction

Driving is a crucial acquired skill, important for both convenient commuting and a sense of independence. Safe driving relies on precise neurobiological processes such as visual acuity and eye movements, hearing, memory, spatial orientation, optimal decision-making, appropriate head and body posture, and coordinated eye-hand or eye-leg movements. However, older adults [1] and individuals with certain neurological conditions, including various movement disorders, often experience compromised driving abilities. Among the latter, patients with movement disorders, both hyperkinetic and hypokinetic, often have compromised driving skills. [2], [3], [4], [5], [6]. To be a safe and effective driver, it is essential to have an accurate insight into one’s driving skills, as high-risk drivers should not be allowed to drive. Patients with any potential physical or mental impairment must accurately assess their driving skills and have use reality checks without overestimating their capabilities. Objective driving assessment (ODA) can provide valuable insights into driving deficits but is not widely available or accessible.

To address this gap, we developed the Baylor Driving Questionnaire for Movement Disorders (BDQMD) which assesses several factors related to driving that may be affected in patients with hyperkinetic and hypokinetic movement disorders. In this pilot study, we aimed to determine if BDQMD scores correlate with the performance on ODA, so that in the future studies the instrument can be further validated as a surrogate marker of driving skills, obviating the need of ODA.

## 2 Methods

This prospective, cross-sectional study was conducted at the Parkinson’s Disease Center and Movement Disorders Clinic (PDCMDC), Baylor College of Medicine, Houston, Texas. Patients diagnosed with any movement disorder at our center were asked if they would be interested in a questionnaire-based assessment (using BDQMD), followed by more objective, “behind-the-wheel”, driving assessment. BDQMD is a 10-item questionnaire and score on each item ranges from 1 to 5 (see supplementary document-1). The BDQMD was developed by the authors based on their clinical experience and published assessments of driving skills. Although we did not utilize the Delphi method, the drafts of the assessments were reviewed and edited by the authors and tested for clarity with patients before the BDQMD was finalized. It was completed by the patients in their caregivers during their clinic encounter.

The ODA was conducted at a certified driving rehabilitation center (Strowmatt Rehabilitation Services, Houston, Texas, USA) where patients are referred on a regular basis. As part of standard of care at the center, participants underwent a comprehensive driving evaluation, which included off-road cognitive testing, 100 video driving scenarios from the driver performance analysis system (DPAS, https://www.dpasnow.com/Information.aspx) and an on-road driving examination conducted in a dual-control vehicle under supervision of a certified driving rehabilitation specialist who rated aspects of the subjects driving. Although participants completed a full clinical on-road driving evaluation, the on-road performance data were not included in the statistical analyses. The DPAS score was used as the primary objective outcome. The decision to rely on DPAS for analysis was made to minimize assessor subjectivity and inter-rater variability during on-road evaluations.

Through the DPAS, various aspects of driving including driver and traffic knowledge, traffic perceptual skills, traffic risk analysis, traffic procedures, and brake reaction time were objectively evaluated. DPAS included viewing 100 video driving scenes and agreeing or disagreeing with a statement regarding each scene within 5 s. Upon completion (approximately 30 min), scores were produced for each of the modules (https://www.dpasnow.com/Information.aspx). All subjects were rated on the above five components of driving. Overall score in the ODA was correlated with the scores on BDQMD.

The study protocol was approved by the Institution Review Board (IRB) of Baylor College of Medicine and all subjects provided written informed consent before their enrollment into the study.

### 2.1 Statistical analysis

We summarized patient characteristics, BDQMD total score, and the individual BDQMD items using mean and standard deviation (SD) for continuous variables or frequency and percentage for categorical variables. These descriptive analyses were done overall for all participants who completed the BDQMD, as well as for the subset who later underwent the ODA. We determined the Spearman’s correlation coefficient between the total BDQMD score with the DPAS score and its components. The correlation of BDQMD with the DPAS total score was the primary outcome measure and the correlation with the DPAS component scores was the exploratory secondary outcomes. Given the limited sample size, analyses of secondary outcomes (i.e., component scores) were not adjusted for multiple comparisons.

## 3 Results

Of the 142 patients who completed the BDQMD, 25 (17.6%) underwent the ODA. There were no significant differences in the age, gender distribution, and duration of disease between the patients completing ODA (n = 25) vs who did not (n = 117). Among those 142 patients who completed BDQMD, majority had PD (n = 91, 64%); the other diagnoses were essential tremor (n = 11, 7.7%), ET-PD overlap (n = 9, 6.3%), Tourette syndrome (n = 12, 8.5%), Huntington’s disease (n = 5, 3.5%), tardive dyskinesia (n = 3, 2.1%), functional movement disorder (n = 3, 2.1%), dystonia (n = 3, 2.1%), hemifacial spasm (n = 2, 1.4%), corticobasal syndrome (n = 1, 0.7%), multiple system atrophy (n = 1, 0.7%), and episodic ataxia (n = 1, 0.7%). The mean total BDQMD score for the whole cohort was 14.7 ± 5.6 (range 10–47). Although the mean total BDQMD was numerically higher in participants who did not go for ODA (15.1 ± 5.9) compared to those who did (12.8 ± 2.9), the difference was not statistically significant (p = 0.065).

**Fig. 1:**
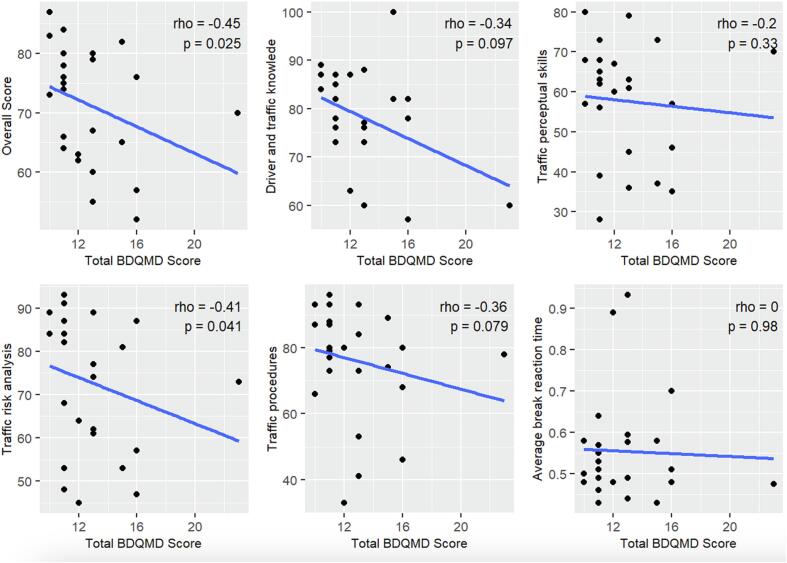
Correlation plots of total score on BDQMD with various components and the total score of driver performance analysis system.

As mentioned above, the primary objective was to explore if performance in ODA correlates with BDQMD. The mean total BDQMD score of the 25 patients who underwent ODA was 12.8 ± 2.9 (Table 1). Based on overall scores on DPAS, 20 patients had average driving skill (total DPAS score 60–80), one was highly skilled (total DPAS score > 80, and 4 had minimum driving skill (total DPAS score < 60). Of the latter, 2 had PD, one had Huntington’s disease, and one had ataxia. BDQMD had significant negative correlation (Fig. 1) with overall DPAS score (r = −0.45, 95% CI −0.72, −0.06, p = 0.025) and traffic risk analysis component (r = −0.41, 95% CI −0.69, −0.02, p = 0.041). (Fig. 1) The results remained significant for correlation analysis adjusted for age and disease duration.

**Table 1:** Summary of 25 patients who underwent objective driving assessment.

**Diagnoses**	PD-14, ET: 4, HD:2, ET-PD: 1, ET-CD-1, EA: 1, OMD: 1, TS-1
**Women**	6/25 (24%)
**Mean age**	66.0 ± 12.5 years
**Years of driving**	49.4 ± 12.7 years
**Mean BDQMD score**	12.8 ± 2.9
**Overall DPAS score**	71.4 ± 9.7
**Mean break reaction time**	0.5 ± 0.1 sec

BDQMD: Baylor driving questionnaire for movement disorders; CD: Cervical dystonia; DPAS: Driver performance analysis system; ET: Essential tremor; HD: Huntington’s disease; OMD: Oromandibular dystonia; PD: Parkinson’s disease; TS: Tourette syndrome.

## 4 Discussion

In this pilot study, 142 patients with various movement disorders completed the 10-item BDQMD and 25 patients (17.6%) underwent behind-the-wheel ODA. The principal finding was a significant negative correlation between the total BDQMD score and the overall DPAS score.

Impairment in driving ability is common among patients with movement and neurodegenerative diseases. [3], [4] In a recent publication, based on a population-based survey involving 540 patients with PD, 80% of whom possessed a driver license, 40% required adjustments in their driving style to compensate for the driving impairment and majority did not discuss the driving impairment with their healthcare providers [2]. Therefore, the availability of an easy-to-administer questionnaire that could reflect driving impairment is crucial. Our study showed negative correlation of BDQMD score with performance in ODA. This is an important first step in bringing the questionnaire to real-world use. While our study does not make BDQMD a proxy for the actual driving assessment, it provides support for developing BDQMD as a useful screening tool for driving impairment.

One of the major hurdles for this kind of study is the patient recruitment for ODA. In this study, only 17% of the patients who completed BDQMD eventually participated in the ODA. While it was not statistically significant, BDQMD score was greater in patients who opted out for ODA. Therefore, there could be some participation bias. In other words, only those who were confident of their driving skills decided to participate. That could be the reason why only 4 out of 25 patients undergoing ODA were found to have minimum driving skills.

Due to this limitation of low sample size along with a clinically heterogenous cohort, it was difficult to reliably predict what score range on the BDQMD would be considered “safe”. Among the 25 patients who underwent ODA, four demonstrated minimal driving skills. A preliminary analysis suggested that a BDQMD score of 13 or higher may be associated with minimal driving ability. However, due to the limited number of patients with minimum driving skills, this finding should be interpreted with caution. Given the limited sample size, analyses of secondary outcomes (i.e., component scores) were not adjusted for multiple comparisons. These secondary analyses were exploratory and hypothesis-generating, and findings should be interpreted accordingly. Additionally, we did not evaluate the classification performance because only four participants showed minimum skills in the DPAS test. Future studies with larger sample sizes are needed to formally assess and validate the classification performance. Larger studies are essential to perform a robust cut-off score analysis and establish a more reliable threshold. Relatively small sample size also did not allow us to compare the differences between hypo and hyperkinetic disorders.

In conclusion, despite some limitations, our study demonstrates a significant correlation between BDQMD scores and DPAS metrics. These results suggest that BDQMD may have value as a screening instrument for driving impairment in individuals with movement disorders. However, further validation in larger and more homogeneous populations is necessary before it can be recommended for routine clinical or real-world application.

## Funding sources

No specific funding was received for this work.

## 6 Financial disclosures for the previous 12 months

**Abhishek Lenka:** The author declares that there are no additional disclosures to report.

**Ruosha Li**: The author declares that there are no additional disclosures to report.

**Alan Gonzalez:** The author declares that there are no additional disclosures to report.

**Rory Mahabir:** The author declares that there are no additional disclosures to report.

**Joseph Jankovic:** The author has the following financial disclosures.

**Research/Training Funding**: Abbott; AbbVie Inc; Amneal Pharmaceuticals, Boston Scientific; CHDI Foundation; Dystonia Coalition; Medtronic Neuroscience; Merz Pharmaceuticals; Michael J Fox Foundation for Parkinson Research; Parkinson’s Foundation.

**Consultant/Advisory Board**: AbbView Inc; Motric Bio.

**Royalties:** Cambridge; Elsevier; Medlink: Neurology; Lippincott Williams and Wilkins; UpToDate; Wiley-Blackwell.

## 7 Ethical compliance statement

• Permission of the Institutional review board was obtained.
• Written informed consent was obtained from the participants.
• We confirm that we have read the Journal’s position on issues involved in ethical publication and affirm that this work is consistent with those guidelines.

## CRediT authorship contribution statement

**Abhishek Lenka:** Writing – original draft, Formal analysis, Data curation. **Ruosha Li:** Writing – review & editing, Formal analysis. **Karim Makhoul:** Conceptualization, Data curation, Writing – review & editing. **Alan Gonzalez:** Writing – review & editing, Data curation. **Rory D. Mahabir:** . **Joseph Jankovic:** Writing – review & editing, Supervision, Project administration, Data curation, Conceptualization.

## Declaration of competing interest

The authors declare that they have no known competing financial interests or personal relationships that could have appeared to influence the work reported in this paper.
