# Supplementary material for: Driving impairment in patients with movement disorders: examining the Baylor driving questionnaire by objective driving assessment
Source: Clin Park Relat Disord. 2026 Feb 10;14:100429. doi: 10.1016/j.prdoa.2026.100429 (PMC12925279; doi:10.1016/j.prdoa.2026.100429)
Supplement: Supplementary Data 1 [file mmc1.docx]

**Supplementary document**

**Baylor Driving Questionnaire for Movement Disorders (BDQMD)**

First Name/Last Name: ______________________________ DOB: _______ Date (M/D/Y):_______

**Please, read each of the following items carefully before selecting the 1-5 response**

1. How often do you notice drowsiness or lapse in your alertness while driving?
   1. Never
   2. At least once every 6 months, but can continue to drive
   3. At least once a month, rarely have to stop driving transiently
   4. At least once a week, frequently have to stop driving
   5. Almost every time I drive as a result of which I have stopped driving
2. How often do you lose control of your hands or arms when trying to control the steering wheel, shifting gears or otherwise maneuver the vehicle?
   1. Never
   2. At least once every 6 months, but can continue to drive
   3. At least once a month, rarely have to stop driving transiently
   4. At least once a week, frequently have to stop driving
   5. Almost every time I drive as a result of which I have stopped driving
3. How often do you experience loss of control of your feet/legs such as missing the break or gas pedal?
   1. Never
   2. At least once every 6 months, but can continue to drive
   3. At least once a month, rarely have to stop driving transiently
   4. At least once a week, frequently have to stop driving
   5. Almost every time I drive as a result of which I have stopped driving
4. How often is your ability to drive impaired because of trouble keeping your eyes open or involuntary eye closure (e.g. blepharospasm)?
   1. Never
   2. At least once every 6 months, but can continue to drive
   3. At least once a month, rarely have to stop driving transiently
   4. At least once a week, frequently have to stop driving
   5. Almost every time I drive as a result of which I have stopped driving
5. How often is your ability to check your blind spot or side view mirrors or turn your head impaired because of limitation of head/neck movement or abnormal head movement or posture (e.g. head tremor, neck tic, cervical dystonia/torticollis)?
   1. Never
   2. At least once every 6 months, but can continue to drive
   3. At least once a month, rarely have to stop driving transiently
   4. At least once a week, frequently have to stop driving
   5. Almost every time I drive as a result of which I have stopped driving
6. How often is your driving impaired because of slowness, stiffness, hand or leg tremor, tics or other involuntary movements?
   1. Never
   2. At least once every 6 months, but can continue to drive
   3. At least once a month, rarely have to stop driving transiently
   4. At least once a week, frequently have to stop driving
   5. Almost every time I drive as a result of which I have stopped driving
7. Do high-speed roads, heavy traffic, bad road/weather conditions, such as darkness, rain or snow, influence your decision or ability to drive?
   1. Never
   2. Slightly. I might need to be slower or more cautious.
   3. Moderately. I can still drive during such conditions, but I am definitely slower and much more cautious.
   4. Extremely difficult. I prefer to avoid driving under such conditions.
   5. I do not drive in such conditions because of my disease.
8. Does your condition affect someone’s driving when you are the passenger?
   1. Never
   2. Slightly. My involuntary movements or noises may possibly distract the driver.
   3. Moderately. My involuntary movements or noises sometimes distract the driver.
   4. Extremely difficult. My involuntary movements or noises often distract the driver.
   5. I am never a passenger because of my involuntary movements or noises.
9. Have you had any difficulty maintaining your vehicle within the lanes when driving?
   1. Never
   2. At least once every 6 months, but can continue to drive
   3. At least once a month, rarely have to stop driving transiently
   4. At least once a week, frequently have to stop driving
   5. Almost every time I drive as a result of which I have stopped driving
10. Have you had any difficulty with missing stop signs or stoplights, or difficulty with turning across oncoming traffic?
    1. Never
    2. At least once every 6 months, but can continue to drive
    3. At least once a month, rarely have to stop driving transiently
    4. At least once a week, frequently have to stop driving
    5. Almost every time I drive.
